# A case of superior mesenteric artery embolism successfully treated by combined thrombus retrieval therapy using the CAPTIVE technique

**DOI:** 10.1093/bjrcr/uaag025

**Published:** 2026-07-08

**Authors:** Tomoki Kawano, Tetsuaki Sugimoto, Maki Imada, Mitsuru Tamura, Takuya Hara, Ichiro Ikushima, Minako Azuma, Yoshiko Okita

**Affiliations:** Department of Neurosurgery, Miyakonojo Medical Association Hospital, Miyakonojo 885-0002, Japan; Department of Neurosurgery, Faculty of Medicine, University of Miyazaki, Miyazaki 889-1692, Japan; Department of Neurosurgery, Miyakonojo Medical Association Hospital, Miyakonojo 885-0002, Japan; Department of Radiology, Miyakonojo Medical Association Hospital, Miyakonojo 885-0002, Japan; Department of Radiology, Faculty of Medicine, University of Miyazaki, Miyazaki 889-1692, Japan; Department of Neurosurgery, Miyakonojo Medical Association Hospital, Miyakonojo 885-0002, Japan; Department of Neurosurgery, Faculty of Medicine, University of Miyazaki, Miyazaki 889-1692, Japan; Department of Radiology, Faculty of Medicine, University of Miyazaki, Miyazaki 889-1692, Japan; Department of Radiology, Miyakonojo Medical Association Hospital, Miyakonojo 885-0002, Japan; Department of Radiology, Faculty of Medicine, University of Miyazaki, Miyazaki 889-1692, Japan; Department of Neurosurgery, Faculty of Medicine, University of Miyazaki, Miyazaki 889-1692, Japan

**Keywords:** superior mesenteric artery, embolism, intestinal necrosis, stent retriever, aspiration catheter, CAPTIVE

## Abstract

Acute superior mesenteric artery (SMA) embolism is potentially fatal if intestinal necrosis develops, and prompt revascularization is essential. Although thrombolytic therapy and aspiration thrombectomy are commonly employed, thrombolysis is contraindicated in patients with hemorrhagic lesions. We report the application of the continuous aspiration prior to intracranial vascular embolectomy (CAPTIVE) technique in an elderly woman with acute SMA embolism complicated by hemorrhagic stroke. Mechanical thrombectomy using a React 71 aspiration catheter and Solitaire stent retriever was performed under proximal balloon flow control. Staged thrombectomy of the jejunal and ileal branches achieved wide recanalization with a limited number of passes, avoiding intestinal necrosis. This case highlights the feasibility of adapting an established neuroendovascular technique to visceral arterial occlusion. The CAPTIVE technique may represent a minimally invasive alternative in selected patients in whom thrombolytic therapy is not indicated.

## Introduction

The continuous aspiration prior to intracranial vascular embolectomy (CAPTIVE) technique is a combined mechanical thrombectomy strategy widely used in neuroendovascular therapy for acute ischemic stroke. By integrating a stent retriever with continuous aspiration through a large-bore catheter, this technique enhances clot engagement and retrieval efficiency while minimizing distal embolization. Its effectiveness and safety have been demonstrated in the management of large vessel occlusions in the cerebral circulation.^1^

Acute superior mesenteric artery (SMA) embolism is a rare but life-threatening condition that can result in intestinal necrosis if not promptly treated. Cardiogenic embolism accounts for approximately 40%-50% of cases.^2^ Early revascularization is essential to prevent bowel infarction and avoid emergent laparotomy. Endovascular therapy has increasingly become the preferred strategy in selected cases without peritonitis; however, thrombolytic therapy may be contraindicated in patients with recent or active bleeding, including hemorrhagic stroke, or in those at high risk of hemorrhagic complications.^3^^,^^4^

In this report, we describe the application of the CAPTIVE technique to SMA embolism with distal embolization in a patient in whom thrombolytic therapy was contraindicated. We also describe technical modifications, including proximal balloon occlusion and staged peripheral branch recanalization, and discuss the feasibility and potential advantages of adapting this neuroendovascular strategy to abdominal interventions.

## Case presentation

### Patient background

The patient was a woman in her 80s with a complaint of abdominal pain. She had a history of atrial fibrillation and hypertension. Magnetic resonance imaging (MRI) revealed a wide infarction in the left posterior inferior cerebellar artery region, which was judged to be a cardiogenic cerebral embolism. Because of hemorrhagic infarction, the patient was followed up without anticoagulation therapy. On the 7th day at 4:00 pm, she complained of epigastric pain.

### Physical examination and blood sample findings

The patient had a body temperature of 36.2 °C, heart rate of 118 bpm, and blood pressure of 178/89 mmHg. JCS I-2 revealed no cerebellar ataxia or evident neurological deficits. Her abdominal findings showed a flat and soft abdomen, with no abdominal mass, tenderness, or muscular defense. Blood sample results revealed a d-dimer level of 26.5 μg/mL, but no other specific findings. Serum lactate level was not measured in this emergency setting.

### Imaging results

Brain MRI on admission demonstrated left cerebellar infarction with hemorrhagic transformation. Because of this hemorrhagic lesion, thrombolytic therapy was contraindicated. Contrast-enhanced CT revealed occlusion of the superior mesenteric artery with reduced enhancement of the small bowel. No CT findings strongly suggestive of irreversible bowel necrosis, such as pneumatosis intestinalis, portal venous gas, mucosal hemorrhage, or hemoperitoneum, were observed (Figure 1).

**Figure 1:**
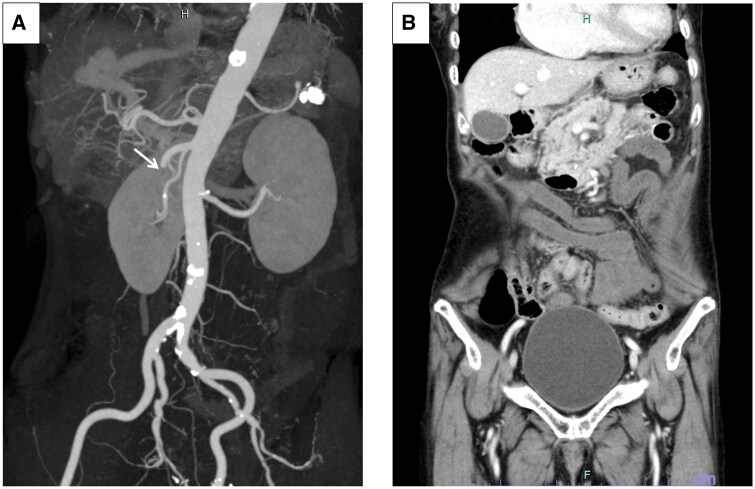
Contrast-enhanced CT findings before intervention. A: CTA/MIP image demonstrating occlusion of the superior mesenteric artery (arrows ). B: Coronal contrast-enhanced CT showing reduced enhancement of the small bowel. No CT findings strongly suggestive of irreversible bowel necrosis, such as pneumatosis intestinalis, portal venous gas, mucosal hemorrhage, or hemoperitoneum, were observed.

Based on these CT findings and her clinical background, SMA embolism of presumed cardioembolic origin was diagnosed.

### Treatment plan

As there were no CT findings strongly suggestive of irreversible bowel necrosis or peritonitis, endovascular treatment was planned.^4^ Urokinase therapy was contraindicated in this patient because of a hemorrhagic stroke.^2^^,^^3^ Considering the large amount of thrombus and distal embolization, the CAPTIVE technique using a combination of a stent retriever and aspiration catheter was planned for thrombus retrieval therapy.^1^^,^^5^

### Surgical procedure

The patient was transferred to the angiography suite approximately 3 hours after symptom onset.

First (Figure 2A and B), a 9-Fr long sheath was inserted via the right femoral artery. Intravenous heparin (2000 U) was administered after sheath placement. Diagnostic angiography using a 4-Fr RC2 catheter confirmed occlusion of the SMA. The system was then exchanged for a 9-Fr Optimo FLEX balloon guiding catheter positioned at the SMA orifice. Balloon inflation enabled proximal flow control, and manual aspiration resulted in retrieval of a large proximal thrombus (Figure 2B). Although the main trunk was recanalized, residual thrombi remained in the first and second jejunal arteries and the peripheral ileal artery (Figure 3B).

**Figure 2:**
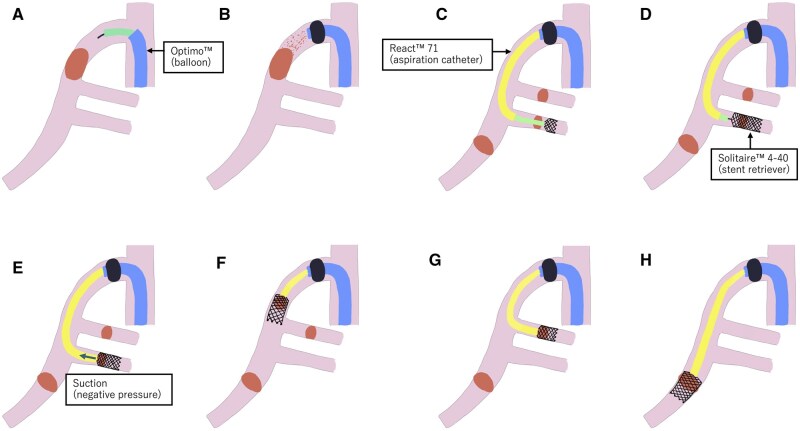
Schematic illustration of the thrombus retrieval procedure using the CAPTIVE technique. A: A 4-Fr RC2 (65 cm) with a 0.035-inch Radifocus guide wire (180 cm) is advanced into the superior mesenteric artery (SMA). After confirming SMA occlusion, the RC2 catheter is removed while the guide wire is left in place. A 9-Fr Optimo FLEX (90 cm) with a 6-Fr JB2 (120 cm) is then advanced to position the Optimo within the SMA. B: The Optimo balloon is inflated to achieve proximal flow control, and aspiration is performed. C, D: To retrieve thrombi dispersed into the first and second jejunal arteries and the peripheral ileal artery, a 0.071-inch React 71 (132 cm) aspiration catheter, a 0.027-inch Phenom 27 (160 cm) microcatheter, and a 0.014-inch ASAHI CHIKAI (200 cm) guidewire are advanced distally. A Solitaire 4 × 40 stent retriever is deployed to engage the thrombus. E: The React 71 catheter is advanced to the proximal surface of the thrombus. F: Continuous aspiration (negative pressure) is applied through the React 71 catheter. When the catheter tip contacts and seals against the thrombus, a negative pressure gradient is generated, allowing en bloc thrombus retrieval using the CAPTIVE technique. G, H: Additional thrombi in the jejunal and ileal branches are removed using the same technique.

**Figure 3:**
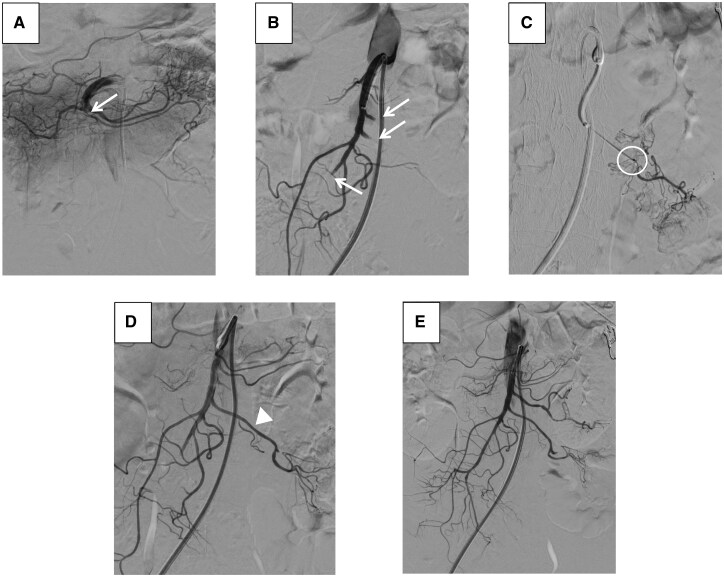
Imaging of the thrombus retrieval procedure using the CAPTIVE technique. A: Angiography via a 4-Fr RC2 catheter demonstrates occlusion at the origin of the superior mesenteric artery (SMA). B: After aspiration with a 9-Fr Optimo, residual embolic occlusion of the first and second jejunal arteries and peripheral ileal branches (arrows) is observed. C: A React 71 and Phenom 27 system is advanced distally, and the microcatheter is navigated beyond the thrombus (circle). D: The CAPTIVE technique is applied to the second jejunal artery, achieving recanalization (arrowhead). E: Final angiography after thrombectomy demonstrates successful recanalization of the SMA and major branches.

Next (Figure 2C–F), a 0.071-inch React 71 aspiration catheter was advanced into the affected peripheral branches over a 0.027-inch Phenom 27 microcatheter and a 0.014-inch ASAHI CHIKAI guidewire. A Solitaire 4 × 40 stent retriever was deployed distal to the thrombus (Figure 2C and D). Continuous aspiration was applied via the React 71 catheter positioned at the proximal face of the clot, generating a negative pressure gradient under proximal balloon flow control (Figure 2E). When the catheter tip engaged and sealed against the thrombus, en bloc retrieval was performed using the CAPTIVE technique (Figure 2F).

Finally (Figure 2G and H), staged thrombectomy was performed in the first jejunal and ileal arteries. Final angiography demonstrated restoration of flow in the major SMA branches without evidence of distal embolization (Figure 3E). Large fresh thrombi were retrieved (Figure 4).

**Figure 4:**
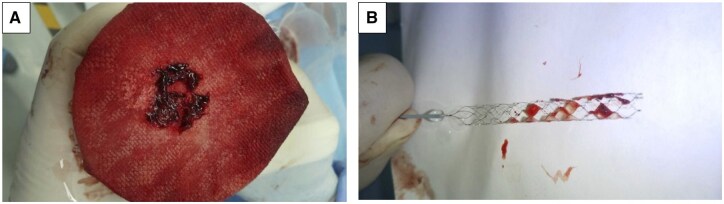
Images of the thrombus post-retrieval. A: Thrombus retrieved from the Optimo (Tokai Medical Products, Japan). A large fresh red thrombus was aspirated. B: Photograph taken immediately after thrombus retrieval using the Solitaire stent retriever (Medtronic, USA) showing thrombus incorporated into the stent.

### Postoperative course

The postoperative course was uneventful. The patient resumed oral intake without evidence of intestinal necrosis and was transferred to a rehabilitation hospital on postoperative day 30.

## Discussion

In the present case, the coexistence of acute cerebral infarction and SMA embolism in a patient with atrial fibrillation strongly suggested a systemic cardioembolic event rather than localized arterial thrombosis.

In this setting, the combined CAPTIVE technique using a stent retriever and aspiration catheter was applied for acute SMA embolism, achieving rapid and extensive reperfusion with a limited number of passes, thereby preventing intestinal necrosis.

Endovascular treatment for SMA embolism commonly includes thrombolytic therapy with urokinase, thrombus aspiration using a large-bore guiding catheter through the brachial or inguinal artery, percutaneous transluminal angioplasty, and stenting.^2–4^ However, in the present case, the patient had a hemorrhagic infarction in the left cerebellar hemisphere; therefore, thrombolytic therapy was contraindicated.^2^^,^^3^ Additionally, because of the large amount of thrombus and high possibility of distal embolization, aspiration alone was deemed inadequate.

Mechanical thrombus retrieval using the CAPTIVE technique was therefore selected, combining a stent retriever and an aspiration catheter as described in neuroendovascular therapy.^1^ Although a similar endovascular approach for SMA embolism has been reported,^5^ the present case incorporated proximal balloon flow control and staged peripheral branch recanalization, which differs from previously described techniques. In contrast to other reports,^6^ after deployment of the stent retriever, the aspiration catheter was advanced to the proximal surface of the thrombus to allow en bloc retrieval. Additionally, a balloon guide catheter was placed proximally to increase suction efficiency. Furthermore, in this case, the device system was advanced into the peripheral jejunal and ileal branches to extend the area of reperfusion. Although distal branch navigation is generally feasible, acute branch angles may reduce catheter stability. In such situations, an intermediate catheter can provide additional distal support, allowing stable device advancement and thrombus retrieval. In the present case, React 71 was used as the intermediate catheter and was also utilized for aspiration.

The mechanical rationale for this configuration lies in the positioning of the aspiration catheter at the proximal face of the thrombus under balloon flow control. By temporarily reducing antegrade flow, proximal balloon occlusion may increase the negative pressure gradient across the clot during aspiration. This hemodynamic environment may enhance en bloc clot retrieval and reduce the risk of distal embolization compared with aspiration alone.^7^^,^^8^

The React 71 has favorable flexibility and trackability and may facilitate access to small peripheral arteries.^9^ The Solitaire stent retriever has an open-cell design and provides stable clot engagement, which may also help maintain system stability during aspiration.^10^ A combination of these characteristics increases the success rate of thrombus retrieval. Notably, the combined use of React 71 and Solitaire facilitated device guidance to the peripheral branches, which was especially useful for the dispersed thrombus in the jejunal and ileal arteries. This combined technique was considered advantageous in a situation that would have been difficult to manage with aspiration alone.

Although the use of a stent retriever for SMA embolism has been reported by Miura et al.^11^ and Tamura et al.,^6^ previous reports have primarily described aspiration alone or single-device thrombectomy. Aspiration-only techniques are technically straightforward; however, repeated passes may be required when the thrombus is organized or large. Conversely, while stent retriever thrombectomy provides strong clot engagement, clot fragmentation and distal embolization may occur during retrieval, particularly in the absence of proximal flow control. To the best of our knowledge, there have been no prior reports describing staged application of the CAPTIVE technique involving multiple peripheral branches under proximal balloon flow control. In the present case, this combined approach was intended to integrate the advantages of both methods while minimizing their respective limitations. This staged recanalization strategy is an important therapeutic tool, particularly in cases involving a pre-necrotic intestinal tract.

However, there were some limitations of this technique. A 9-Fr sheath was required; therefore, the brachial approach was practically difficult and limited to the inguinal artery. Additionally, catheter exchange was necessary to guide the catheter using a balloon, which is a complicated procedure. Furthermore, at present, the use of a stent retriever and aspiration catheter for the endovascular treatment of abdominal vessels is not covered by insurance, which is another drawback for the clinical application of this procedure.

Further case reports of this combined technique for SMA embolism, and an evaluation of its effectiveness and safety through prospective registries and multicenter studies, may result in its standardization and insurance coverage. The reporting of similar cases will be the first step toward establishing treatment guidelines in the future.

## Conclusion

The combined technique (CAPTIVE) is a minimally invasive and effective option, especially in patients at a high risk of bleeding and in whom thrombolytic therapy is contraindicated. The peripheral arterial approach using the characteristics of the React 71 and Solitaire devices is expected to improve recanalization rates. The usefulness and indications of this combined technique for SMA embolism should be further investigated with the accumulation of similar cases and through studies verifying its treatment outcomes.

## Learning points

- Acute superior mesenteric artery (SMA) embolism without peritonitis can be managed with prompt CT angiography–based diagnosis and early endovascular revascularization, potentially avoiding emergent laparotomy.
- In patients with SMA embolism and recent hemorrhagic stroke, the CAPTIVE technique using a stent retriever and large-bore aspiration catheter provides a minimally invasive alternative when thrombolytic therapy is contraindicated.
- The combination of a React 71 aspiration catheter and a Solitaire stent retriever enabled navigation into the jejunal and ileal branches and en bloc retrieval of distal emboli, achieving wide reperfusion with only a small number of passes.
- The use of a balloon guiding catheter in the proximal SMA may enhance aspiration efficiency and reduce the risk of distal embolization during mesenteric mechanical thrombectomy.
